# Coadministration of calcium chloride with lead acetate can improve motility of cauda epididymal spermatozoa in Swiss white mice

**Published:** 2016-02

**Authors:** Farhad Golshan Iranpour, Soleiman Kheiri

**Affiliations:** 1 *Department of Anatomical Sciences, Isfahan University of Medical Sciences, Isfahan, Iran.*; 2 *Herbal Medicine Research Center, Shahrekord University of Medical Sciences, Shahrekord, Iran.*

**Keywords:** *Calcium*, *Lead*, *Mouse*, *Sperm motility*

## Abstract

**Background:**

Lead is an industrial heavy metal that can decrease sperm motility.

**Objective:**

The aim was to investigate the protective effects of calcium against lead on motility of spermatozoa.

**Materials and Methods:**

In total 40 adult male Swiss white mice were randomly divided into 5 groups (control, lead of 1^st^ wk, lead of 2^nd^ wk, lead/calcium of 1^st ^wk and lead/calcium of 2^nd^ wk). The lead groups of mice were injected by a single dose of lead acetate (200 mg/kg) intraperitoneally. Lead/calcium groups of mice were injected by a single same dose of lead acetate along with three doses of 80 mg/kg calcium chloride. The control group of mice was injected only with same volume of distilled water through the same route. Mice of 1^st^ and 2^nd^ wk groups were sacrificed through cervical dislocation one and two weeks after injections respectively.

**Results:**

Mean of the progressive motile spermatozoa of cauda epididymis in lead/calcium group of the first week was higher than the lead group of the first week and this difference was significant. There was not any significant difference among weight of testes and epididymides of all groups.

**Conclusion:**

It can be concluded that calcium can decrease the effects of lead on sperm motility.

## Introduction

Lead, mercury, chromium, nickel and cadmium are examples of heavy metals. Among these metals lead is the most famous one. In addition to workers who deal with lead, other people are also exposed to lead pollution daily. Lead excretion is slow in the body and biological half-life of it is 24-40 days (1). In recent years, the negative effects of environmental substances on male genitalia have been received considerable interest. Lead could reduce the number of sperms and decrease follicle stimulating hormone (FSH), but the amount of luteinizing hormone (LH) was not changed (2). 

The effects of low or moderate doses of lead acetate were investigated on rats for 24 wk. The results showed that there was no change in body weight and weight of testes and epididymides (3). Ca²+ has an important role in many cellular functions of sperm such as flagellar beating, movement velocity and acrosome reaction. For appropriate function, motile sperm maintains intracellular free calcium concentration (4). Because of the crucial role of calcium in sperm cell function and motility, it may protect sperm against environmental factors. So the aim of this study was to investigate the protective effect of calcium against lead on motility of cauda epididymal spermatozoa of mouse.

## Materials and methods

**Animals**

In this prospective study, 40 healthy Swiss white male mice obtained from Pasteur’s Institute (Tehran, Iran) were used. Their weights were between 25-40 gr. Every five mice were kept in a cage for two weeks in animal house to acclimate to laboratory conditions. Food and water was provided ad libitum. All animal procedures were approved by Shahrekord University of Medical Sciences Ethical Committee, Shahrekord, Iran.

**Chemicals**

Purified lead acetate and calcium chloride were purchased from Merck (Merck Company, Germany). Also Ham’s F10 was purchased from Gibco (Gibco Company, England). Lead acetate and calcium chloride doses of 200 mg/kg and 80 mg/kg were selected according to the previous studies of Achyra *et al* and Aboul-Ela (5, 6). Both of them were dissolved in distilled water and injected intraperitoneally.

**Experimental design**

40 adult male Swiss white mice were randomly selected and divided into 5 groups (control, lead of 1^st^ wk, lead of 2^nd^ wk, lead/calcium of 1^st^ wk and lead/calcium of 2^nd^ wk). Lead groups were injected by a single dose of 200 mg/kg of lead acetate. Lead/calcium groups were injected by a single dose of 200 mg/kg lead acetate followed by three injections of 80 mg/kg calcium chloride. Calcium chloride injection was done one time immediately after injection of lead acetate and two times in two days following the first injection. 

Mice of the 1^st^ and 2^nd^ wk groups were sacrificed through cervical dislocation one and two weeks after lead injection respectively. The control group was also injected with the same volume of distilled water. This group was sacrificed one week after the injection of distilled water. All injections in all groups were intraperitoneally.

**Weighing of testes and epididymides**

Right side testes and epididymides of the mice were removed, cleaned of accessory tissues and weighed in grams.

**Sperm motility**

Sperm suspension was prepared by squeezing of the tail part of epididymides in Ham’s F10 medium. After putting one drop of sperm suspension on a slide, a cover slip was placed on it to assess percentage of progressive motile spermatozoa. Then the slides were examined under the 40× objective lens of light microscope. Three slide replicates were assessed and at least 200 sperms were counted and the percentage of progressive motile sperms was calculated for each slide. 

**Statistical analysis**

The data was analyzed using Statistical Package for Social Studies (SPSS) software. Testes and epididymides weight and sperm motility were reported as mean±SD for each group. An analysis of the variance was performed for weight. One way ANOVA and post hoc analysis were carried out by LSD for motility of spermatozoa. A comparison is considered significantly different when p<0.05.

## Results

**Weight of testes and epididymides**

Statistical analysis of the results obtained from weighing of the testes and epididymides showed that there is no significant difference among various groups of study (respectively p=0.11, p=0.31, Table I).

**Sperm motility**

The mean percentages of progressive motile sperms were measured in each group. In order to ensure the normal distribution of data, primarily Kolmogorov-Smironov test was performed. Based on this test the distribution of sperm motility percentages were normal in different groups (p=0.2). Therefore, these variables were compared in groups of one-way ANOVA with LSD post hoc test. The results for motility are given in table I. As it can be seen in the table I, the mean percentages of progressive motile sperms in lead and lead/calcium groups were significantly lower than the control group (for all of them p<0.001).

Also the mean percentage of progressive motile spermatozoa in lead /calcium group of the first week was higher than the lead group of the first week and this difference was significant (p=0.04). Moreover, the results indicated that there was no significant difference between the percentages of motility in the lead/calcium group of the second week and the lead group of the second week (p=0.46).

**Table I:** Percentage of motile spermatozoa of cauda epididymis and weight of right testis and epididymis in control, lead and lead/calcium groups

	**Motility (%)**	**Weight of testis (g)**	**Weight epididymis (g)**
Control group	65.3 ± 3.58	0.220 ± 0.016	0.023 ± 0.002
Lead of 1^st^ week	21.6 ± 7.7 ͣ	0.224 ± 0.020	0.026 ± 0.001
Lead of 2^nd^ week	39.7 ± 4.1 ͣ ᵇ	0.206 ± 0.044	0.027 ± 0.004
Lead/calcium of 1^st^ week	32.2 ± 15.5 ͣ ᵇ	0.185 ± 0.060	0.030 ± 0.006
Lead/ calcium of 2^nd^ week	43 ± 8.1 ͣ ͨ	0.230 ± 0.032	0.031 ± 0.002

Data are presented as mean±SD.

## Discussion

In our preceding report, we demonstrated that lead reduces motility and viability of spermatozoa but this effect will disappear up to three weeks after the injection. In contrast to motility and viability, lead does not denature DNA of sperms (7). Because the effects of lead will disappear three weeks after the lead injection, in the present study the results were followed for two weeks. Significant changes were not seen in the weight of testes and epididymides of mice of lead group in the present study. These results are in accordance with those of some researchers who did not find any significant changes in the weight of testis and seminal vesicle after administration of lead in rats (2, 3). Nevertheless, some researchers reported that lead administration could reduce weight of mice testes (6). It seems that lead dosage and route of administration are the main reasons for the difference in the results.

Our results show that mean percentage of progressive motile spermatozoa in lead/calcium group of the first week was significantly higher than the lead group of the first week and this difference was significant. It has been shown that certain vitamins like vitamin C and E have the ability to decrease the toxic effects of lead on sperm count and morphology in Swiss white mice (8). Moreover, the protective function of zirconium against the clastogenic effects of lead has been reported (9). Prescribed calcium supplementation during pregnancy reduces the amount of lead in the blood and may decrease it in maternal and subsequently fetal blood (10). Rising of dietary calcium decreases lead absorption (11). A number of studies have revealed that intracellular calcium plays an important role in sperm motility and different steps of fertilization, such as, capacitation, acrosome reaction and zona penetration (12, 13). However, the exact role of intracellular calcium in spermatogenesis is unclear (13).

It has been illustrated that calcium at lower concentrations (lower than 10 µg) promoted motility and velocity of spermatozoa (14). Aboul- Ela demonstrated the protective effect of calcium against genotoxicity of lead in somatic and germ cells (6). These findings are consistent with those of Weber *et al* study. They found that verapamil, that is a calcium antagonist, increases DNA damage which is induced by Bleomicin (15). To our knowledge, our results are the first ones that indicate co-administration of calcium with lead could reduce the effects of lead on the sperm motility up to one week. However, this effect is temporary and did not continue in the second week. The results of this study complete the results of Aboul-Ela that indicate co-administration of calcium with lead reduces chromosomal changes of spermatocytes (6). It seems that re-injection of calcium after one week can avoid the toxic effects of lead even in the second week. This issue needs further research. 

It can be concluded that calcium can decrease the potential effects of lead on reducing sperm motility in caudal part of epididymis of mouse.
